# Elevated Cardiac Troponin Levels as a Predictor of Increased Mortality Risk in Non-Cardiac Critically Ill Patients Admitted to a Medical Intensive Care Unit

**DOI:** 10.3390/jcm13206025

**Published:** 2024-10-10

**Authors:** Turkay Akbas

**Affiliations:** Division of Intensive Medicine, Department of Internal Medicine, School of Medicine, Düzce University, Düzce 81620, Turkey; turkayakbas@yahoo.com

**Keywords:** Troponin I (TnI), medical intensive care unit (MICU), short-term mortality, long-term mortality, SOFA scores, APACHE II scores

## Abstract

**Background**: Cardiac troponin I (TnI) is a specific marker of myocardial damage used in the diagnosis of acute coronary syndrome (ACS). TnI levels can also be elevated in patients without ACS, which is linked to a worse prognosis and mortality. We evaluated the clinical implications and prognostic significance of serum TnI levels in critically ill non-cardiac patients admitted to the intensive care unit (ICU) at a tertiary-level hospital. **Materials and Methods**: A three-year retrospective study including the years 2017–2020 was conducted to evaluate in-hospital mortality during ICU stay and mortality rates at 28 and 90 days, as well as one and two years after admission, in 557 patients admitted to the medical ICU for non-cardiac causes. **Results**: TnI levels were elevated in 206 (36.9%) patients. Patients with elevated TnI levels were significantly older and had higher rates of comorbidities, including chronic heart failure, coronary heart disease, and chronic kidney disease (*p* < 0.05 for all). Patients with elevated TnI levels required more invasive mechanical ventilation, vasopressor infusion, and dialysis in the ICU and experienced more shock within the first 72 h (*p* = 0.001 for all). High TnI levels were associated with higher Acute Physiological and Chronic Health Evaluation (APACHE) II (27.6 vs. 20.3, *p* = 0.001) and Sequential Organ Failure assessment (8.8 vs. 5.26, *p* = 0.001) scores. Elevated TnI levels were associated with higher mortality rates at 28 days (58.3% vs. 19.4%), 90 days (69.9% vs. 35.0%), one year (78.6% vs. 46.2%), and two years (82.5% vs. 55.6%) (*p* < 0.001 for all). Univariate logistic regression analysis revealed that high TnI levels were a strong independent predictor of mortality at all time points: **28 days** (OR = 1.2, 95% CI: 1.108–1.3, *p* < 0.001), **90 days** (OR = 1.207, 95% CI: 1.095–1.33, *p* = 0.001), **one year** (OR = 1.164, 95% CI: 1.059–1.28, *p* = 0.002), and **two year** (OR = 1.119, 95% CI: 1.026–1.22, *p* = 0.011). Multivariate analysis revealed that age, albumin level, APACHE II score, and requirements for dialysis and vasopressor use in the ICU were important predictors of mortality across all timeframes, but elevated TnI levels were not. **Conclusions**: Elevated TnI levels in critically ill non-cardiac patients are markers of disease severity. While elevated TnI levels were significant predictors of mortality in the univariate analysis, they lost significance in the multivariate model when adjusted for other factors. Patients with elevated TnI levels had higher mortality rates across all timeframes, from 28 days to two years.

## 1. Introduction

Cardiac troponins, including troponin I (TnI) and troponin T (TnT), are structural proteins released into circulation following myocardial injury. TnI is more specific for cardiac muscle damage and is widely used in the diagnosis of acute coronary syndromes (ACSs). However, elevated levels of troponins can also be seen in critically ill patients without primary cardiac disease, often due to conditions such as sepsis, renal failure, and hypoxia, which cause myocardial strain. These non-ischemic causes of troponin elevation may represent type II myocardial infarction (MI), which is characterized by supply–demand mismatch and myocardial injury without coronary artery occlusion. The presence of elevated levels in patients with severe illnesses presents an interpretative challenge. Studies in the ICU and among high-risk surgical patients have consistently found a strong correlation between high troponin levels and increased rates of in-hospital and long-term mortality [1,2]. This has spurred interest in the use of troponins for risk evaluation during major surgeries, unrelated to cardiac issues. For instance, the Vascular Events in Non-cardiac Surgery Patients Cohort Evaluation (VISION) study observed a notable link between elevated postoperative troponin T levels and 30-day mortality, even when accounting for various patient factors and surgical types [3]. However, the exact reasons for troponin elevation and its direct relationship with mortality remain elusive. The causes of troponin release are diverse, and the specific contributions of inflammatory and ischemic myocardial damage are still debated. Moreover, evidence regarding whether troponin can independently predict in-hospital mortality in critically ill patients, considering the severity of the illness, is mixed [2].

Implementing complex risk prediction models that incorporate factors such as the diagnosis and severity of illness and other patient-specific characteristics is a common practice in intensive care to standardize procedures and results across different units. The Acute Physiological and Chronic Health Evaluation (APACHE) II model employed by the Scottish Intensive Care Society Audit Group integrates acute physiological abnormalities, initial diagnoses, chronic health conditions, and patient age [4]. Given the well-established association between troponin level and mortality, particularly in the context of cardiovascular events, its role as a predictor of all-cause mortality warrants further exploration. In existing risk prediction frameworks, the inclusion of TnI could potentially enhance predictive accuracy, not only for cardiac mortality but also for broader outcomes.

This present study aimed to evaluate the independent relationship between early TnI levels and short-term (28 days and 90 days) and long-term mortality (1 year and 2 years) among patients admitted to the medical ICU (MICU). By examining TnI in this context, we sought to determine its value as a robust predictor across a range of mortality outcomes in critically ill patients.

## 2. Materials and Methods

### 2.1. Study Design and Patients

This study was designed as a three-year cross-sectional retrospective study that included data from January 2017 to December to 2020. We focused on evaluating in-hospital mortality as well as subsequent mortality at 28 and 90 days and at one- and two-years post-admission for patients admitted to the MICU. The follow-up included monitoring patients after hospital discharge with mortality data, including date, tracked through the Ministry of Health system to ensure a comprehensive two-year post-discharge follow-up. There was no loss during the follow-up.

### 2.2. Inclusion and Exclusion Criteria

**Figure 1:**
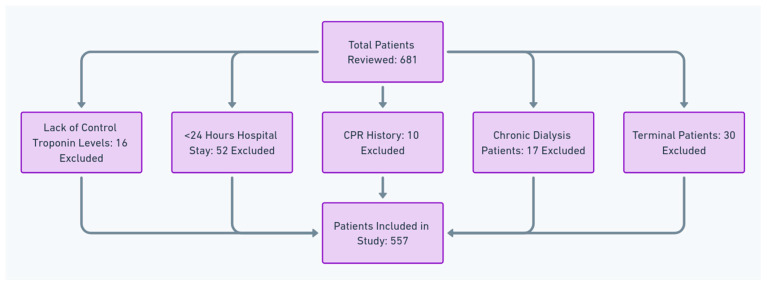
Patients’ exclusion flowchart.

As depicted in Figure 1, the study population consisted of patients (aged ≥ 18 year-old) admitted to the MICU. To qualify for inclusion, patients had to be free from acute coronary syndromes, as this study aimed to investigate the non-cardiac causes of troponin I elevation.

Patients were excluded based on the following criteria:

- Lack of control troponin levels: 16 excluded.
- Hospital stay < 24 h: 52 excluded.
- Cardiopulmonary resuscitation (CPR) history: 10 excluded.
- Chronic dialysis patients: 17 excluded.
- Terminal patients: 30 excluded.

Ultimately, from an initial pool of 681 patients, 557 met the inclusion criteria and were included in the study.

### 2.3. Data Collection

Data were gathered within the first 24 h of admission to the MICU. Patient information was collected from the hospital record systems and the patient information database, which included post-discharge follow-up data.

### 2.4. Patient Data

Patient data were collected within the first 24 h of ICU admission. This included demographic information, clinical profiles, and specific details, such as TnI level, admission diagnoses, initial APACHE II, and Sequential Organ Failure Assessment (SOFA) scores. Subsequent outcomes tracked included the need for invasive mechanical ventilation (IMV), all types of shock within the first 72 h, dialysis requirement during ICU stay, acute kidney injury (AKI) development, vasopressor requirements during ICU stay, and mortality rates at various time points after admission (28 days, 90 days, 1 year, and 2 years). The collected data also encompassed biochemical parameters at admission and during the ICU stay, along with hospitalization parameters, including ICU and hospital stay duration [4]. Troponin I levels were categorized as low (<0.16 ng/mL) and elevated (≥0.16 ng/mL), with the threshold of 0.16 ng/mL used to define elevated levels for the purposes of this study. TnI levels were quantitatively measured using the Elecsys Troponin I STAT COBAS kit (Roche Diagnostics, Basel, Switzerland) on a COBAS e411 analyzer.

### 2.5. Ethical Considerations

Ethical approval for this study was obtained from the Düzce University School of Medicine Ethical Committee (date: 21 November 2022, number: 2022/187), which ensured that all the research was conducted in accordance with the highest ethical standards. No data that distinguished any of the patients’ privacy were used. Written informed consent was not required owing to the retrospective nature of the study.

### 2.6. Statistical Analysis

Data analysis focused on the relationship between early TnI levels and hospital mortality. Univariate and multivariate analyses were conducted to identify risk factors for two-year mortality among patients with elevated early TnI levels at MICU admission. The normal-distributed data were presented in mean (±standard deviation [SD]), the non-normally distributed variables were presented in median (interquartile range [IQ 25–75]), and the categorical variables were presented in number (%). The association between TnI levels and illness severity was assessed by comparing SOFA and APACHE II scores between patients with elevated TnI levels (≥0.16 ng/mL) and those with lower levels (<0.16 ng/mL) using the Independent Groups *t*-Test.

Multivariate Analysis and Validation:

We conducted multivariate logistic regression analyses to identify independent predictors of mortality at different time points (28-day, 90-day, 1-year, and 2-year mortality). Variables included in the multivariate models were selected based on their clinical relevance and statistical significance in the univariate analyses (*p* < 0.05). Covariates such as age, comorbidities (CHF, chronic kidney disease, etc.), APACHE II score, SOFA score, vasopressor use, and the need for invasive mechanical ventilation were adjusted for in the models. The models were internally validated using cross-validation techniques to assess the stability and predictive accuracy of the models. We evaluated the goodness-of-fit of each model using the Hosmer–Lemeshow test, ensuring that regression assumptions (linearity, homoscedasticity, and multicollinearity) were satisfied to ensure the robustness of the findings. The regression models were validated using internal validation techniques, including cross-validation, to assess the stability and predictive power of the models. Statistical significance was determined using tests, including the Chi-Square Test, Independent Groups *t*-Test, and Mann–Whitney U Test. Kaplan–Meier analysis was employed for survival analysis, and Receiver Operating Characteristic (ROC) curves were generated to evaluate the prognostic accuracy of TnI levels for different mortality timeframes, with Area Under Curve (AUC) values. The *p* < 0.05 was accepted as significant.

## 3. Results

This study assessed the demographic and clinical profiles of patients with non-coronary diseases-related troponin levels. Of the patients evaluated, 274 (49.2%) were female and 283 (50.8%) were male (*p* = 0.682). Regarding TnI levels, 351 patients (63%) had low levels (<0.16 ng/mL) while 206 patients (37%) exhibited high levels (≥0.16 ng/mL).

### 3.1. Demographic and Clinical Profile of Patients with Non-Coronary Cardiac Troponin Levels

The medical history showed that hypertension (HT) was present in 376 patients (67.5%), diabetes mellitus (DM) in 185 patients (33.2%), chronic heart failure (CHF) in 203 patients (36.4%), cerebrovascular disease (CVD) in 113 patients (20.3%), chronic kidney disease (CKD) in 99 patients (17.8%), coronary artery disease (CAD) in 144 patients (25.9%), and chronic obstructive pulmonary disease (COPD) in 119 patients (21.4%).

The patients were admitted for a variety of diagnoses. Gastrointestinal diseases accounted for 23 admissions (4.1%), cardiovascular diseases excluding CAD for 29 (5.2%), non-pneumonia pulmonary diseases for 61 (11%), sepsis (all systemic infection) for 251 (45.1%), neurological diseases for 69 (12.4%), and postoperative follow-up for 99 (17.8%). Other reasons were cited in 25 patients (4.4%).

Upon admission, 271 patients (48.7%) visited the emergency room while 286 (51.3%) were admitted through other services. IMV was required in 283 patients (50.8%). The average APACHE score within the first 24 h was 23.1 ± 8.9 and the SOFA score was 6.6 ± 4.1. Additionally, within 72 h of admission, shock was observed in 180 patients (32.3%).

### 3.2. Clinical Characteristics and Mortality Outcomes Based on Troponin Levels

In the assessment of patients with varying levels of TnI, patients with higher troponin levels (≥0.16 ng/mL) were older on average (73.1 years) than those with lower levels (<0.16 ng/mL) with an averaged 69.1 years (*p* = 0.003). The history of CKD (24.8% vs. 13.7%, *p* = 0.001), CHF (48.1% vs. 32.2%, *p* = 0.007), and CAD (32.0% vs. 22.2%, *p* = 0.011) were more pronounced in patients with higher troponin levels than in those with lower levels. This group also demonstrated a greater need for IMV (70.4%), experienced shock within the first 72 h of admission (52.4%), and required more dialysis in the ICU (31.1%) and vasopressor infusion (77.2%), all of which were statistically significant compared with their counterparts with lower troponin levels (*p* = 0.001 for each comparison).

Higher TnI levels were found to be associated with significantly increased SOFA and APACHE II scores in the first 24 h; the means were 8.8 ± 4.1 and 27.6 ± 8.5, respectively, in the high troponin group, compared with 5.26 ± 3.3 and 20.3 ± 8.2, respectively, in the low troponin group (*p* = 0.001 for both scores). Biochemical parameters revealed significant differences in albumin, creatinine, C-reactive protein, the PaO_2_/FiO_2_ ratios, lactate levels, white blood cell counts, and platelet counts, indicating more severe clinical presentations in the high troponin group. These patients had longer ICU stay durations (*p* = 0.010) (Table 1).

### 3.3. Mortality Impact of Troponin Levels at Various Post-ICU Admission Intervals

Over different timeframes, mortality outcomes varied significantly with TnI level. Within 28 days of ICU admission, patients with high TnI levels had a mortality rate of 58.3%, compared with 19.4% among those with low levels, with statistical significance (*p* < 0.001). The mortality rate at 90 days showed a similar pattern: 69.9% of patients with high TnI levels died versus 35.0% in the low troponin group (*p* < 0.001).

This trend extended to the one-year mark, where 78.6% of patients with high TnI levels passed away as opposed to 46.2% with low levels (*p* < 0.001). At the two-year evaluation, 82.5% of patients with elevated TnI levels died, in contrast to 55.6% of those with lower levels, maintaining the pattern of significant difference (*p* < 0.001).

The cumulative mortality over the two years was 65.5% for the total patient population, indicating a considerable impact of TnI levels on long-term survival. These findings were further substantiated through the Kaplan–Meier analysis, emphasizing the prognostic significance of troponin levels in ICU patients (Table 2).

### 3.4. Determination of Troponin I Cut-Off Values for Predicting Mortality

The prognostic utility of TnI was quantified via identifying cut-off values that correlated with mortality at various intervals after ICU admission. The AUC for 28-day mortality was 0.76, with an optimal cut-off value of 0.171 ng/mL, demonstrating a sensitivity of 63.3% and specificity of 76.7% (*p* < 0.001). For 90-day mortality, the AUC was slightly lower at 0.72, with a cut-off of 0.081 ng/mL, yielding a sensitivity of 68.2% and a specificity of 63.4% (*p* < 0.001).

**Figure 2:**
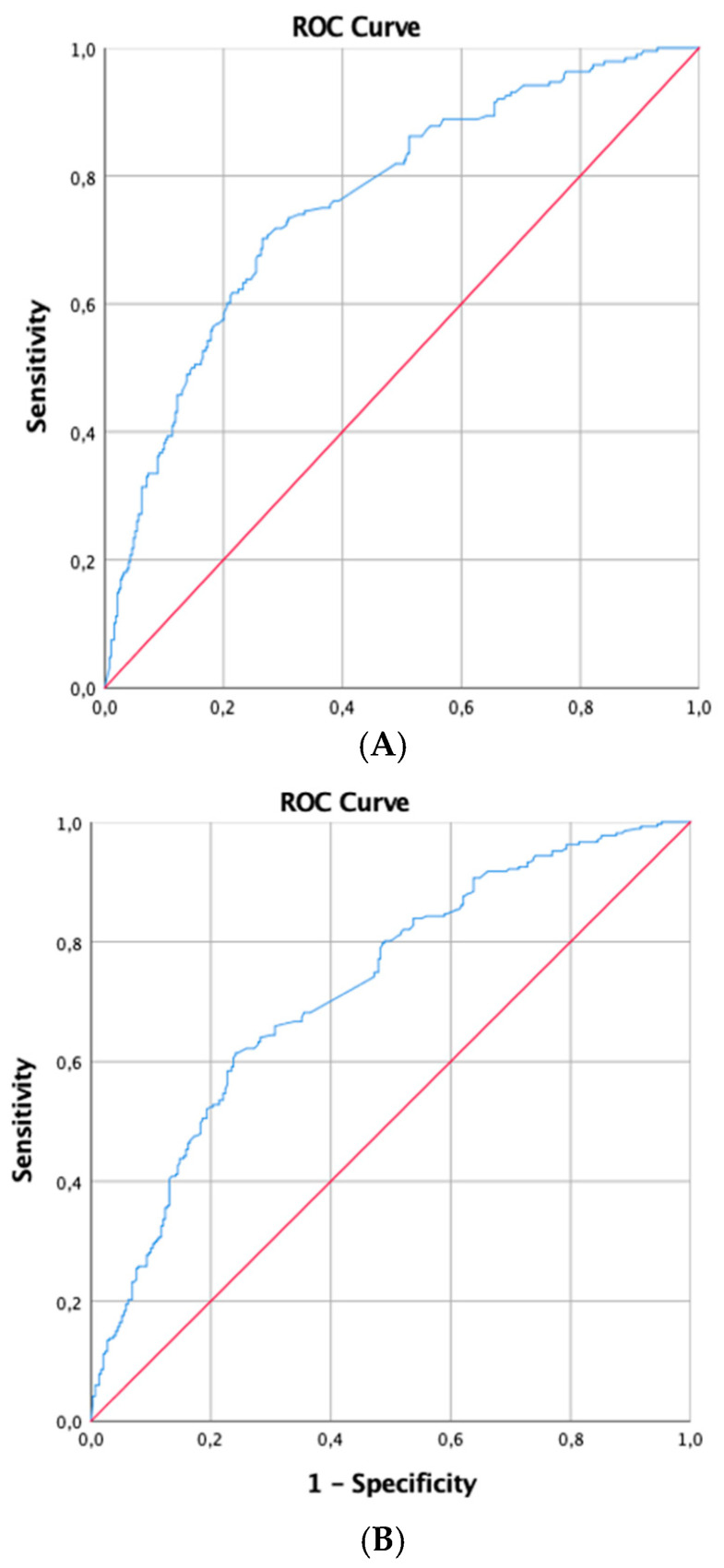

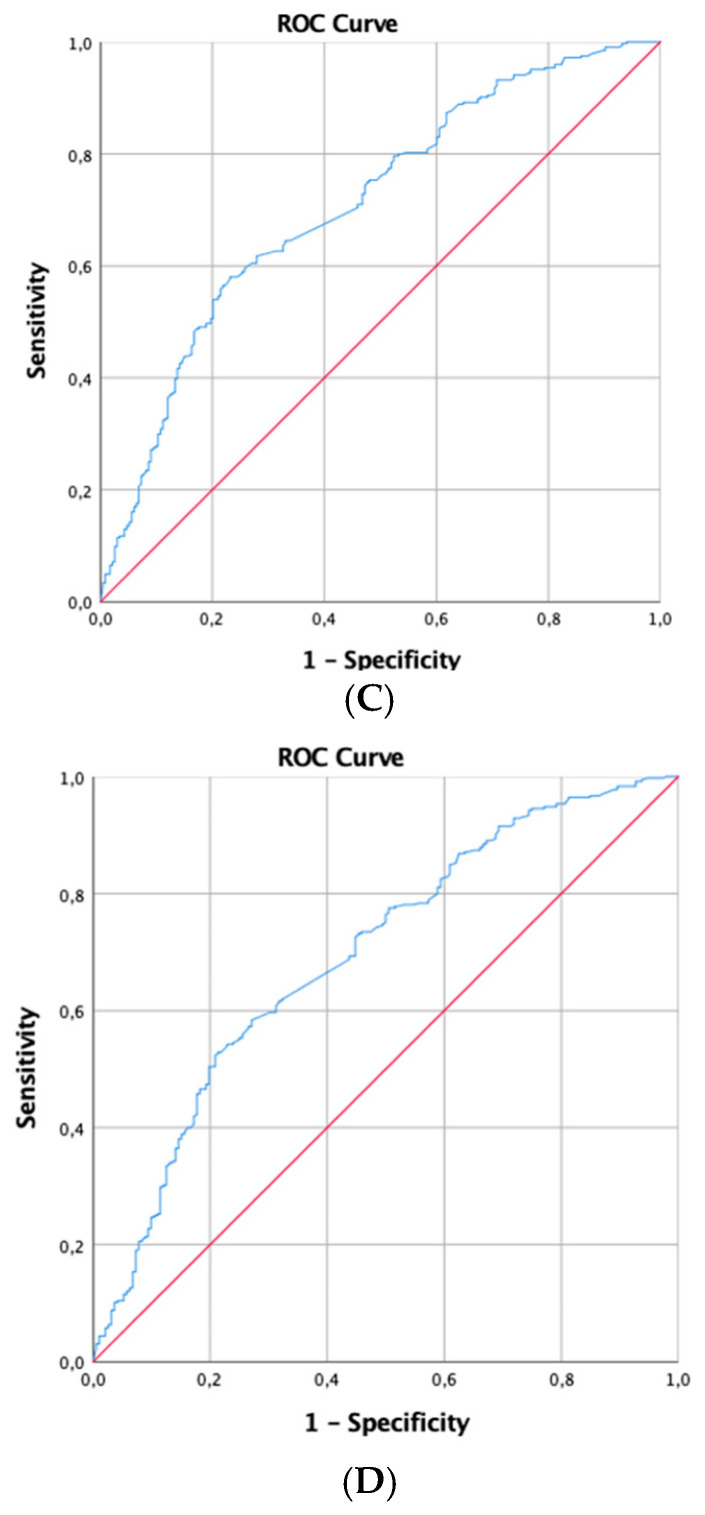
(**A**) ROC curves for the 28 motality; (**B**) ROC curves for the 90 days mortality; (**C**) ROC curves for the 1-year motality; (**D**) ROC curves for the 2-years motality.

At the one-year mark, the AUC was 0.71 and the cut-off value was set at 0.082 ng/mL, with sensitivity and specificity rates of 64.5% and 66.5%, respectively (*p* < 0.001). Finally, for two-year mortality prediction, the AUC stood at 0.70 with the same cut-off value of 0.081 ng/mL, which predicted long-term mortality with a sensitivity of 61.9% and specificity of 67.7% (*p* < 0.001) (Table 3, Figure 2A–D).

### 3.5. Key Predictors of Two-Year Mortality in MICU Patients

In the analysis of two-year mortality risk factors among patients in the MICU, several variables displayed significant associations. Age was a consistent predictor of mortality across all periods, with the odds of death increasing by 2.4% to 5.6% for each additional year of age.

The need for IMV stood out as a strong predictor, with patients requiring IMV having more than seven times the odds of mortality at 28 days compared with those who did not require it (univariate OR: 7.004 [95% CI: 4.627–10.584], *p* < 0.001). This relationship remained significant across all timeframes in univariate analyses, with odds ratios of 5.227 (95% CI: 3.64–7.506, *p* = 0.001) at 90 days, 5.132 (95% CI: 3.555–7.408, *p* < 0.001) at 1 year, and 5.013 (95% CI: 3.406–7.379, *p* < 0.001) at 2 years. However, the effect size decreased when adjusted for other factors in the multivariate analysis, yielding an OR of 2.462 (95% CI: 1.424–4.258, *p* = 0.001) at 28 days and not remaining significant as the timeframes extended to 9 days, 1 year, and 2 years.

The requirement for dialysis in the ICU significantly increased the risk of two-year mortality. This association remained substantial even after adjusting for other factors, with a multivariate odds ratio indicating that patients requiring dialysis had four times the risk of mortality at the two-year follow-up (OR: 4.338, 95% CI: 1.786–10.541, *p* = 0.001). The requirement of vasopressor infusion in the ICU stay was another significant predictor of increased two-year mortality in the univariate and multivariate analyses. In the univariate analysis, patients receiving higher doses had over ten times the odds of two-year mortality (OR: 10.348, 95% CI: 3.373–31.746, *p* < 0.001). APACHE II score in the first 24 h was a significant predictor of two-year mortality, with consistent findings in both univariate and multivariate analyses. Each one-point increase in the APACHE II score corresponded to a 9% increase in the risk of two-year mortality (OR: 1.09, 95% CI: 1.044–1.138, *p* < 0.001).

Elevated TnI levels were significantly associated with higher mortality rates at all observed time points, with the highest risk observed within the first 28 days in the univariate analysis; patients with higher TnI levels had a 58.3% mortality rate at 28 days compared with 19.4% in those with lower levels. This trend continued with a 69.9% mortality rate at 90 days, 78.6% at one year, and 82.5% at two years for patients with elevated TnI levels (*p* < 0.001 for all). However, elevated TnI levels lost its significance as a mortality predictor in the multivariate analysis when adjusted for other factors (Table 4).

## 4. Discussion

In this present study, we found that elevated TnI levels were associated with both short- and long-term mortality in a general MICU patient population. Older patients tended to have higher TnI levels than younger patients. Sex distribution did not show a significant difference between the groups, suggesting that age, rather than sex, may be a more critical factor in TnI elevation, contrary to the Nord-Trøndelag Health Study (HUNT) study that investigated the relationship between TnI levels and sex (male) for cardiovascular disease risk in the general population [5]. Sex, along with TnI elevation, is reported to be an independent risk factor for in-hospital mortality in sepsis patients [6] but not in MICU patients in short- and long-term mortality studies [2].

Patients with high TnI levels had higher incidences of CHF, CKD, and CAD, suggesting that elevated TnI levels are associated with severe underlying cardiovascular and renal conditions in patients admitted to the MICU. Patients with high TnI levels were more likely to present with shock within 72 h of admission, develop AKI in the MICU, and require dialysis, indicating a more severe clinical course in these patients, which was further supported by the higher requirements for IMV and vasopressors. Both SOFA and APACHE II scores were significantly higher in the high TnI group of MICU patients, suggesting that higher TnI levels correlate with greater physiological instability and a higher risk of death. Significant differences were also observed in biochemical parameters, including albumin, creatinine, PaO_2_/FiO_2_ ratio, lactate, white blood cells, and C-reactive protein, indicating more severe metabolic and inflammatory responses in patients with higher TnI levels. A recent study by Babuin et al. was the most comprehensive cohort study conducted before our study. Their work, involving 929 MICU patients, followed the risk factors and mortality over two years, similar to our study [2]. Their findings also highlight how baseline chronic conditions contribute to both short- and long-term mortality, reaffirming the critical importance of early identification and management of these comorbidities to improve patient outcomes [2]. Another study on the association between TnI levels and hospital mortality by Docherty et al. reported that elevated TnI and APACHE II scores were the most relevant parameters for hospital mortality prediction [7].

Overall, short- and long-term mortality were associated with elevated TnI levels, although the mortality rate tended to decrease throughout the timeline. The cut-off point of TnI levels for 28-day mortality stayed high, and even low TnI levels were predictive of mortality at 90-day and longer mortalities. Parameters including age, IMV requirement, shock developed within 72 h of admission, dialysis requirement in the MICU, vasopressor requirement in the MICU, SOFA, and APACHE II scores, and lactate and albumin levels consistently predicted worse outcomes across all time points. Elevated TnI levels due to postoperative follow-up or after a new surgery were generally not associated with worse outcomes across all time points. However, age, vasopressor requirement in the MICU, APACHE II score for the first 24 h, and low albumin levels independently predicted severity and worse outcomes across all time points via multivariate analysis.

Our study revealed that high TnI levels consistently related with mortality at all observed time points, up to two years in univariate analyses. The highest mortality rate occurred within the first 28 days, and progressively decreased thereafter. For patients in the MICU, a high initial TnI level should be considered an important early warning indicator within the first 28 days. These findings can assist in stratifying patients based on their mortality risk, potentially guiding more aggressive or tailored treatment strategies for those with elevated TnI levels. Cardiac troponins are structural proteins found in the myofibrils of cardiomyocytes. The development of high-sensitivity assays for TnI has improved the ability to detect low levels of circulating cardiac troponins, which are often present in individuals with common cardiac conditions and risk factors who have not yet developed cardiovascular disease. Through lowering the detection threshold of troponin assays, the potential use of cardiac troponins has expanded from being a diagnostic tool in the setting of acute coronary syndrome to a biomarker for risk stratification in individuals without known cardiovascular disease [8]. Before our study, Roberts et al. demonstrated that a TnI level of 0.04 ng/mL was associated with hospital mortality in ICU patients [9]. However, their study had some limitations: while it involved a large cohort of consecutively admitted ICU patients, the study did not capture detailed data on admission diagnoses or document the presence of preexisting ischemic heart disease or associated risk factors [9].

Age, along with elevated TnI levels, and consistent statistical significance of age across all timeframes indicates that older patients are at a higher risk of mortality. This is supported by previous studies in critically ill populations, where aging was linked to multiple organ dysfunction and a reduced ability to recover [10,11]. Comorbidities, including CHF, were significant predictors of mortality in the univariate analyses across all timeframes. This finding aligns with previous studies that emphasize the importance of early management of CHF in ICU patients to improve outcomes. However, CHF did not remain significant in the multivariate analyses, indicating that other factors may play a more dominant role when adjusted for. CHF is a well-established predictor of mortality, and our study aligns with other studies that emphasize the early management of CHF in ICU patients to improve outcomes [12,13,14,15]. CKD was particularly significant in predicting long-term mortality (1-year and 2-year) in the univariate analyses. However, CKD did not remain significant in the multivariate analyses, suggesting that its impact may be influenced by other variables when considered together. This is consistent with prior research showing that CKD leads to persistent inflammatory responses and poor metabolic control, worsening prognosis [11,16].

Studies have shown that detectable levels of cardiac troponins are associated with an increased incidence of CAD, CHF, and cardiovascular mortality [17]. According to an ARIC study, TnI < 1.3 ng/dL is a pragmatic biomarker for risk assessment in middle-aged and older adults [18]. We determined even lowered cut-off value as 0.08 ng/dL for long-term mortality in MICU patients. As far as we can report, this is the first reported threshold for the independent risk mortality in MICU patients in the literature. The predictive strength of TnI for mortality demonstrated a robust capability, although it showed a slight decline over time. The cut-off values for TnI were lower for long-term outcomes (0.081 ng/mL for both 90-day and 2-year mortality) compared with a higher threshold for 28-day mortality (0.171 ng/mL), suggesting that even lower levels of TnI may still signify significant long-term risks. The AUC values confirmed that TnI was a reliable mortality predictor with good sensitivity and specificity at various time points. Specifically, the highest sensitivity was observed for 90-day mortality (68.2%), and the highest specificity for 28-day mortality (76.7%). These findings highlight the necessity for nuanced clinical judgment when interpreting TnI levels tailored to individual patient scenarios.

Elevated TnI levels in non-cardiac critically ill patients are often the result of myocardial injury triggered by a variety of systemic conditions. These include sepsis, shock, renal failure, and hypoxia, all of which can cause myocardial strain even in the absence of ACS. Several studies have demonstrated that elevated TnI is a reliable marker of myocardial damage beyond ACS. Myocardial injury may occur due to direct ischemia from microvascular dysfunction or from inflammation caused by systemic conditions like sepsis. Wu et al. demonstrated that elevated troponin I levels were associated with higher mortality and multiorgan failure in non-cardiac patients, even after adjusting for APACHE II scores [1]. Similarly, Babuin et al. showed that TnI elevation in non-cardiac patients was an independent predictor of both short- and long-term mortality [2]. These findings underscore the complex interplay between cardiovascular and systemic illness, where non-ischemic myocardial damage plays a significant role.

In our study, elevated TnI levels were significantly associated with older age, CHF, and CKD, conditions that exacerbate myocardial strain. Although left ventricular ejection fraction (LVEF) was not available in our dataset, chronic heart failure itself was a prevalent comorbidity, as noted in our analysis. Elevated TnI levels in these patients likely reflect underlying subclinical myocardial injury due to these comorbidities, which is consistent with previous research that links CHF and CKD with increased TnI levels and mortality.

High odds ratios in univariate analysis for clinical features such as IMV indicate a strong correlation between IMV use and mortality. The need for IMV reflects significant respiratory failure, which has consistently been associated with adverse outcomes. Shock within 72 h of admission was consistently significant in the univariate analyses across all timeframes, indicating its powerful influence on mortality. This aligns with the existing literature that shock, often due to septic or cardiogenic causes, requires rapid and aggressive intervention to reduce mortality risk [6]. The requirement for dialysis remained significant throughout the study period. AKI and its associated renal replacement therapy are widely documented as adverse prognostic indicators in the ICU [19].

The strong correlation between high SOFA scores and mortality in the univariate supports its utility as a predictor of multiple organ failure, reflecting physiological instability that worsens outcomes [20]. The APACHE II score was significant across all timeframes in both the univariate and multivariate analyses, indicating its robust predictive value. Higher scores reflect greater illness severity, consistent with mortality [21]. Low albumin levels at admission are significant across timeframes and analyses, reinforcing their role as markers of nutritional status, inflammation, and overall health [13,22]. Consistent with the significant lactate elevation in univariate and multivariate analyses, elevated lactate levels indicate metabolic stress and poor tissue perfusion, which are known to be linked to increased mortality [23,24]. These findings are consistent with the literature, emphasizing that a comprehensive approach that considers patient demographics, comorbidities, clinical presentation, and biochemical data are crucial for accurately predicting and managing mortality risks.

Patients undergoing major surgical procedures, especially those admitted to the ICU, are often at increased risk for type II MI due to the significant physiological stress experienced during and after surgery. Factors such as hypoperfusion, hypoxemia, and hemodynamic instability can create an oxygen supply–demand mismatch, leading to myocardial strain without the presence of primary coronary artery disease. This phenomenon has been well-documented in various surgical settings, where elevated troponin levels are commonly observed postoperatively. Recent studies have reinforced the link between elevated high-sensitivity cardiac troponin levels and adverse outcomes, even in non-cardiac patients. Our findings support this, emphasizing the need for careful monitoring and early intervention in patients with elevated troponin levels, particularly following elective surgeries [25].

Clinically, these insights should inform the early identification and targeted treatment plans for critically ill patients. In non-cardiac critically ill patients, elevated TnI levels, even at relatively low thresholds, should be considered a marker of increased mortality risk. These patients may benefit from closer hemodynamic monitoring and early aggressive interventions. Management strategies could include routine serial measurements of TnI to detect trends in myocardial strain, even in the absence of acute coronary syndrome. Furthermore, elevated TnI levels should prompt clinicians to evaluate for underlying causes of myocardial injury, such as sepsis, renal dysfunction, or hypoxia, and initiate prompt treatment for these conditions. Since elevated TnI levels have been linked with long-term adverse outcomes, post-discharge follow-up should be considered for these patients, focusing on preventing rehospitalizations and monitoring for late-onset cardiovascular complications. Implementing these measures can improve both short- and long-term outcomes for patients with elevated TnI in the intensive care setting.

### Study Limitations

Our study has several limitations that should be acknowledged. The study is retrospective, relying on existing hospital records and databases. This design inherently carries a risk of missing or incomplete data, which may affect the results and their generalizability. Data were collected from a single tertiary-level hospital, which may limit the applicability of our findings to other settings, particularly those with different patient demographics or healthcare practices.

Despite these limitations, our study provides valuable insights into the prognostic significance of elevated TnI levels in critically ill patients, emphasizing the need for further prospective, multicenter studies to validate and extend these findings.

## 5. Conclusions

Elevated TnI levels are a significant predictor of short- and long-term mortality in critically ill non-cardiac patients admitted to the MICU. Our study demonstrates that elevated TnI levels correlate with worse outcomes across all time points, with the highest mortality observed within the first 28 days. The findings emphasize the importance of early risk stratification and targeted management strategies for patients with elevated TnI. Even relatively low TnI levels indicate a significant long-term risk, highlighting the value of incorporating TnI into clinical assessment models. Further multicenter studies are needed to confirm these results and explore optimal intervention strategies.

## Figures and Tables

**Table 1:** Comparison of clinical characteristics and outcomes between patients with low and high troponin levels.

	Troponin < 0.16 ng/mL (*n* = 351)	Troponin > 0.16 ng/mL (*n* = 206)	*p*-Value ***
Patient Age (Mean ± SD)	69.1 ± 16.3	73.1 ± 14.4	0.003
Male	176 (50.1%)	107 (51.9%)	0.682
Service at Admission			
Emergency Room	169 (48.1%)	102 (49.5%)	0.755
Clinical Services	182 (51.9%)	104 (50.5%)	
HT	234 (66.7%)	142 (68.9%)	0.582
DM	108 (30.8%)	77 (37.4%)	0.110
CHF	113 (32.2%)	90 (48.1%)	0.007
CVD	69 (19.7%)	44 (21.4%)	0.63
CKD	48 (13.7%)	51 (24.8%)	0.001
CAD	78 (22.2%)	66 (32.0%)	0.011
COPD	74 (21.1%)	45 (21.8%)	0.832
Admission Diagnoses			
Gastrointestinal Diseases	10 (2.8%)	13 (6.3%)	0.047
Cardiovascular Diseases *	15 (4.3%)	14 (6.7%)	0.196
Non-Pneumonia Pulmonary Diseases	39 (11.1%)	22 (10.7%)	0.875
Sepsis (Infection)	142 (40.5%)	109 (52.9%)	0.004
Neurological Diseases	45 (12.8%)	24 (11.7%)	0.686
Postoperative Follow-Up	85 (24.2%)	14 (6.8%)	0.001
Other Reasons **	15 (4.3%)	10 (4.9%)	0.749
Clinical Features and Staging			
Need for IMV	138 (39.3%)	145 (70.4%)	0.001
Shock (Within 72 h of Admission)	72 (20.5%)	108 (52.4%)	0.001
AKI Development in ICU	125 (35.6%)	132 (64.1%)	0.001
Vasopressor Requirement in ICU	140 (39.9%)	159 (77.2%)	0.001
SOFA Score First 24 h (Mean ± SD)	5.26 ± 3.3	8.8 ± 4.1	0.001
APACHE II Score First 24 h (Mean ± SD)	20.3 ± 8.2	27.6 ± 8.5	0.001
Biochemical Parameters at Admission			
Albumin, g/dL (Mean ± SD)	3.1 ± 0.7	2.9 ± 0.7	0.006
Glucose (mg/dL) (IQR 25–75)	146 (118–185)	153 (122.8–200.3)	0.050
Creatinine, (mg/dL) (IQR 25–75)	0.9 (0.7–1.4)	1.6 (1.0–3.1)	0.001
PaO_2_/FiO_2_ (IQR 25–75)	248.6 (167.5–355)	216 (148.6–310.5)	0.001
Hemoglobin (g/dL) (Mean ± SD)	11.1 ± 2.4	10.9 ± 2.4	0.287
PCO_2_ (mm Hg) (IQR 25–75)	37.5 (31.3–45.8)	35.4 (28.8–42.7)	0.045
Lactate (mmol/L) (IQR 25–75)	1.5 (1.1–2.4)	2.2 (1.5–3.5)	0.001
WBC (×10^3^/µL) (IQR 25–75)	10.9 (7.7–15.6)	12.4 (9.5–16.3)	0.017
Platelets (×10^3^/µL) (IQR 25–75)	223,000 (166,000–297,000)	192,000 (143,750–281,500)	0.015
CRP (mg/dL) (IQR 25–75)	6.5 (1.2–13.5)	9.6 (3.6–18.3)	0.001
Treatment and Hospitalization Parameters			
Dialysis Requirement in ICU	45 (12.8%)	64 (31.1%)	0.001
Vasopressor Duration (Days) (IQR 25–75)	5 (2–9.8)	5 (2.3–13.4)	0.104
MICU Stay Duration (Days) (IQR 25–75)	6 (3–12)	6 (4–17)	0.010
Hospital Stay Duration (Days) (IQR 25–75)	11 (7–20)	10 (4.8–20)	0.138

AKI: acute kidney injury, APACHE: Acute Physiology and Chronic Health Evaluation, CAD: coronary artery disease, CHF: chronic heart failure, CKD: chronic kidney disease, COPD: chronic obstructive pulmonary disease, CRP: C-reactive protein, CVD: cerebrovascular disease, DM: diabetes mellitus, HT: hypertension, IQR: interquartile range, IMV: invasive mechanical ventilation, PaO_2_/FiO_2_: partial pressure of arterial oxygen to fraction of inspired oxygen ratio, MICU: medical intensive care unit, PCO_2_: partial pressure of carbon dioxide, SD: standard deviation, SOFA: Sequential Organ Failure Assessment, WBC: white blood cell. * CAD: except acute myocardial ischemia. ** Other reasons: hypernatremia (4 patients), intoxication (3 patients), hypervolemia (3 patients), hyperosmolar coma (3 patients), anaphylactic shock (3 patients), diabetic ketoacidosis (2 patients), road accident (2 patients), acute kidney injury (2 patients), alcohol intoxication (1 patient), anemia (1 patient), conversion (1 patient). *** Chi-Square Test, Independent Groups *t*-Test, Mann–Whitney U Test.

**Table 2:** Comparative mortality outcomes based on troponin level thresholds over time.

Timeframe	Troponin Level	Deceased (*n*)	(%)	Surviving (*n*)	(%)	Total (*n*)	*p*-Value
28 Days	Low	68	19.4	283	80.6	351	<0.001
	High	120	58.3	86	41.7	206	
	Total	188	33.8	369	66.2	557	
90 Days	Low	123	35.0	228	65.0	351	<0.001
	High	144	69.9	62	30.0	206	
	Total	267	47.9	290	52.1	557	
1 Year	Low	162	46.2	189	53.8	351	<0.001
	High	162	78.6	44	21.4	206	
	Total	324	58.2	233	41.8	557	
2 Years	Low	195	55.6	156	44.4	351	<0.001
	High	170	82.5	36	17.5	206	
	Total	365	65.5	192	34.5	557	

Kaplan–Meier analysis.

**Table 3:** Troponin cut-off values according to mortality time with asymptotic 95% confidence intervals.

Mortality Time	AUC	Cut-Off	Lower Bound	Upper Bound	Sensitivity	Specificity	*p*-Value
28-Day Mortality	0.76	0.171	0.72	0.80	63.3%	76.7%	<0.001
90-Day Mortality	0.72	0.081	0.68	0.77	68.2%	63.4%	<0.001
1-Year Mortality	0.71	0.082	0.67	0.75	64.5%	66.5%	<0.001
2-Year Mortality	0.70	0.081	0.65	0.74	61.9%	67.7%	<0.001

AUC: area under curve.

**Table 4:** Risk factors for two-year mortality in hospitalized patients: univariate and multivariate analyses.

Variable	Univariate OR (95% CI)	*p*-Value	Multivariate OR (95% CI)	*p*-Value	Univariate OR (95% CI)	*p*-Value	Multivariate OR (95% CI)	*p*-Value
	28-day Parameters				90-day Parameters			
Age	1.021 (1.008–1.034)	0.001	1.024 (1.006–1.042)	0.009	1.03 (1.018–1.042)	0.001	1.036 (1.019–1.053)	<0.001
DM	1.357 (0.939–1.962)	0.104			1.353 (0.95–1.927)	0.094		
CHF	1.755 (1.223–2.519)	0.002	1.254 (0.755–2.08)	0.382	1.631 (1.152–2.308)	0.006	0.969 (0.588–1.595)	0.951
CKD	1.744 (1.12–2.716)	0.014	1.053 (0.553–2.006)	0.875	1.38 (0.892–2.135)	0.148	0.796 (0.413–1.535)	0.496
CAD	1.59 (1.075–2.352)	0.020	1.482 (0.88–2.495)	0.139	1.299 (0.888–1.9)	0.177	1.024 (0.613–1.711)	0.927
GI Diseases	2.667 (1.147–6.202)	0.023			2.098 (0.875–5.032)	0.097		
Sepsis (Infection) Admission	2.064 (1.445–2.948)	<0.001	0.669 (0.389–1.151)	0.146	2.629 (1.865–3.707)	0.001	0.886 (0.524–1.497)	0.651
Postoperative Follow-Up/New Surgery	0.294 (0.165–0.526)	<0.001	0.547 (0.236–1.267)	0.159	0.321 (0.198–0.52)	0.001	0.553 (0.261–1.169)	0.121
IMV Requirement	7.004 (7.004–4.627)	<0.001	2.462 (1.424–4.258)	0.001	5.227 (3.64–7.506)	0.001	1.475 (0.875–2.486)	0.145
Shock (Within 72 h of Admission)	3.811 (2.614–5.556)	<0.001			3.905 (2.668–5.716)	0.001		
Dialysis Requirement in ICU	5.02 (3.218–7.829)	<0.001	2.941 (1.592–5.431)	0.001	4.58 (2.837–7.395)	0.001	3.453 (1.762–6.77)	<0.001
AKI in ICU	3.562 (2.46–5.158)	<0.001			2.975 (2.106–4.203)	0.001		
Vasopressor Requirement in ICU	9.981 (6.3081–15.794)	<0.001	3.163 (1.805–5.543)	<0.001	9.357 (6.33–13.83)	0.001	3.447 (2.092–5.678)	<0.001
SOFA Score First 24 h	1.352 (1.2739–1.43)	<0.001			1.368 (1.288–1.454)	0.001		
APACHE II Score First 24 h	1.128 (1.09–1.15)	<0.001	1.021 (1.004–1.058)	0.005	1.141 (1.112–1.171)	0.001	1.056 (1.018–1.096)	0.003
Albumin	0.427 (0.321–0.568)	<0.001	0.566 (0.382–0.839)	0.005	0.348 (0.262–0.462)	0.001	0.43 (0.291–0.636)	<0.001
HCO3	0.919 (0.889–0.95)	<0.001			0.946 (0.918–0.975)	0.001		
PCO_2_	0.981 (0.968–0.994)	0.005			0.986 (0.974–0.998)	0.018		
Lactate	1.411 (1.264–1.576)	<0.001	1.225 (1.075–1.395)	0.002	1.421 (1.257–1.607)	0.001	1.195 (1.033–1.384)	0.017
WBC	1.027 (1.002–1.052)	0.032			1.025 (1.001–1.049)	0.045		
CRP	1.035 (1.019–1.051)	<0.001	1.014 (0.992–1.036)	0.214	1.036 (1.02–1.052)	0.001	0.998 (0.976–1.02)	0.845
ICU Stay (days)	0.981 (0.969–0.994)	0.005			1.008 (0.999–1.017)	0.101		
Glucose	1.001 (0.999–1.004)	0.278			1.002 (1–1.005)	0.08		
Creatinine	1.384 (1.221–1.569)	<0.001	0.995 (0.841–1.176)	0.951	1.332 (1.169–1.517)	0.001	0.905 (0.759–1.08)	0.269
PaO_2_/FiO_2_	0.998 (0.996–0.999)	0.003			0.998 (0.996–0.999)	0.001		
Troponin I	1.2 (1.108–1.3)	<0.001	1.066 (0.987–1.151)	0.106	1.207 (1.095–1.33)	0.001	1.047 (0.957–1.145)	0.319
	1-yeaar Parameters				2-year Parameters			
Age	1.037 (1.025–1.05)	<0.001	1.048 (1.03–1.066)	<0.001	1.045 (1.032–1.058)	<0.001	1.056 (1.038–1.075)	<0.001
DM	1.371 (0.955–1.97)	0.087			1.378 (0.943–2.013)	0.097		
CHF	1.984 (1.381–2.85)	<0.001	0.986 (0.577–1.683)	0.957	2.214 (1.504–3.261)	<0.001	0.946 (0.538–1.663)	0.846
CKD	2.055 (1.278–3.302)	0.003	1.283 (0.622–2.647)	0.505	2.376 (1.404–4.019)	0.001	1.667 (0.76–3.658)	0.203
CAD	1.38 (0.933–2.042)	0.107	1.035 (0.599–1.787)	0.903	1.165 (0.778–1.745)	0.459	0.73 (0.413–1.293)	0.281
GI Diseases	2.095 (0.813–5.398)	0.126			1.94 (0.709–5.309)	0.197		
Sepsis (Infection) Admission	2.852 (2–4.067)	<0.001	0.719 (0.411–1.256)	0.246	3.228 (2.209–4.716)	<0.001	0.88 (0.491–1.576)	0.667
Postoperative Follow-Up/New Surgery	0.257 (0.161–0.409)	<0.001	0.344 (0.163–0.724)	0.005	0.263 (0.168–0.413)	<0.001	0.402 (0.196–0.824)	0.013
IMV Requirement	5.132 (3.555–7.408)	<0.001	1.368 (0.781–2.396)	0.273	5.013 (3.406–7.379)	<0.001	1.341 (0.74–2.43)	0.333
Shock (Within 72 h of Admission)	4.004 (2.654–6.039)	<0.001			4.93 (3.085–7.879)	<0.001		
Dialysis Requirement in ICU	5.94 (3.34–10.562)	<0.001	4.825 (2.157–10.792)	<0.001	5.429 (2.895–10.182)	<0.001	4.338 (1.786–10.541)	0.001
AKI in ICU	3.286 (2.298–4.697)	<0.001			2.976 (2.049–4.324)	<0.001		
Vasopressor Requirement in ICU	9.113 (6.171–13.456)	<0.001	3.591 (2.118–6.088)	<0.001	8.88 (5.88–13.411)	<0.001	3.754 (2.117–6.658)	<0.001
SOFA Score First 24 h	1.38 (1.295–1.47)	<0.001			1.39 (1.299–1.488)	<0.001		
APACHE II Score First 24 h	1.162 (1.129–1.195)	<0.001	1.074 (1.032–1.118)	<0.001	1.169 (1.135–1.205)	<0.001	1.09 (1.044–1.138)	<0.001
Albumin	0.328 (0.245–0.438)	<0.001	0.367 (0.241–0.56)	<0.001	0.386 (0.289–0.515)	<0.001	0.507 (0.331–0.777)	0.002
HCO3	0.963 (0.935–0.992)	0.013			0.974 (0.945–1.004)	0.087		
PCO_2_	0.987 (0.976–0.998)	0.023			0.99 (0.979–1.001)	0.064		
Lactate	1.398 (1.225–1.595)	<0.001	1.174 (0.991–1.391)	0.063	1.402 (1.213–1.621)	<0.001	1.187 (0.979–1.441)	0.082
WBC	1.017 (0.992–1.042)	0.181			1.023 (0.996–1.049)	0.092		
CRP	1.039 (1.022–1.057)	<0.001	0.996 (0.972–1.019)	0.713	1.039 (1.02–1.058)	<0.001	0.995 (0.971–1.021)	0.717
ICU Stay (days)	1.057 (1.036–1.079)	<0.001			1.09 (1.058–1.122)	<0.001		
Glucose	1.002 (1–1.005)	0.063			1.001 (0.999–1.004)	0.303		
Creatinine	1.462 (1.248–1.711)	<0.001	0.861 (0.714–1.038)	0.117	1.457 (1.227–1.73)	<0.001	0.823 (0.676–1.003)	0.053
PaO_2_/FiO_2_	1.462 (1.248–1.711)	<0.001			0.997 (0.996–0.999)	<0.001		
Troponin I	1.164 (1.059–1.28)	0.002	0.991 (0.906–1.084)	0.842	1.119 (1.026–1.22)	0.011	0.944 (0.867–1.028)	0.184

OR: odds ratio, CI: confidence interval, GI: gastrointestinal, DM: diabetes mellitus, CHF: chronic heart failure, CKD: chronic kidney disease, CAD: coronary artery disease, ICU: intensive care unit, SOFA: Sequential Organ Failure Assessment, WBC: white blood cell, CRP: C-reactive protein, PaO_2_/FiO_2_: partial pressure of oxygen/fraction of inspired oxygen.

## Data Availability

Data are available upon reasonable request from the corresponding author.
